# COVID-19 vaccine hesitancy in Zambia: a glimpse at the possible challenges ahead for COVID-19 vaccination rollout in sub-Saharan Africa

**DOI:** 10.1080/21645515.2021.1948784

**Published:** 2021-07-06

**Authors:** Andrea C. Carcelen, Christine Prosperi, Simon Mutembo, Gershom Chongwe, Francis D. Mwansa, Phillimon Ndubani, Edgar Simulundu, Innocent Chilumba, Gloria Musukwa, Phil Thuma, Kelvin Kapungu, Mutinta Hamahuwa, Irene Mutale, Amy Winter, William J. Moss, Shaun A. Truelove

**Affiliations:** a Department of International Health, International Vaccine Access Center, Johns Hopkins Bloomberg School of Public Health, Baltimore, MD, USA; b Laboratory Science, Macha Research Trust, Choma, Zambia; c Department of Immunology, Tropical Diseases Research Center, Ndola, Zambia; d Ministry of the Health, Government of the Republic of Zambia, Lusaka, Zambia; e Department of Epidemiology, Johns Hopkins Bloomberg School of Public Health, Baltimore, MD, USA; f W Harry Feinstone Department of Molecular Microbiology and Immunology, Johns Hopkins Bloomberg School of Public Health, Baltimore, MD, USA

**Keywords:** COVID-19, vaccine, vaccine safety, vaccine acceptance, vaccine hesitancy, Zambia

## Abstract

With unprecedented speed, multiple vaccines against SARS-CoV-2 are available 1 year after the COVID-19 pandemic was first identified. As we push to achieve global control through these new vaccines, old challenges present themselves, including cold-chain storage, the logistics of mass vaccination, and vaccine hesitancy. Understanding how much hesitancy toward COVID-19 vaccines might occur and what factors may be driving these concerns can improve the ability of public health workers and communicators to maximize vaccine uptake. We nested a survey within a measles-rubella mass vaccination campaign in Zambia in November 2020 and asked about sentiments and beliefs toward COVID-19 and COVID-19 vaccines. Among parents bringing their children to receive a measles-rubella vaccine, we found high acceptability of COVID-19 vaccination of their children, but substantial uncertainty and hesitancy about receiving the vaccine themselves. COVID-19 vaccination hesitancy was correlated with beliefs around COVID-19 severity and risk, as well as vaccine safety and effectiveness.

## Introduction

Zambia will begin administering COVID-19 vaccines in April 2021.^1^ However, the uptake of COVID-19 vaccine remains unknown for Zambia. In developed countries, up to half of the adult population may delay vaccination or refuse COVID-19 vaccines for themselves or their children.^2–4^ In other global contexts, acceptance of a COVID-19 vaccine remains unclear – one study across 19 countries found vaccine acceptance ranged from 55% to 89%, with 65% in Nigeria and 82% in South Africa.^5^ This variability in acceptance may be a result of both historical vaccination acceptance and perceptions of risk and safety from COVID-19 disease and vaccine.

Zambia has a strong childhood vaccination program with a high vaccine uptake; 93% coverage for the first dose of measles-rubella vaccine (MR) in 2019.^6^ The Zambian Ministry of Health also has a long history of successful mass vaccination campaigns reaching over 3 million children with MR vaccines during Child Health Week in November 2020. However, it is unclear whether this will translate to acceptance of novel vaccines targeted to adults, for whom vaccines remain limited to only tetanus toxoids for pregnant women, human papillomavirus, hepatitis B vaccine, and oral cholera vaccine for high-risk residential areas (OCV).

Furthermore, perceived risk among many Zambians may limit acceptance of COVID-19 vaccine. At the time of this study, Zambia had not been hit severely by COVID-19 pandemic, reporting just 1,300 cases and 7 deaths by November 2020, when this study was conducted.^7^ While this has increased to 86,779 reported cases and 1,185 deaths as of 23 March 2021, this still represents only 0.5% of the population. Among populations with limited impact from the pandemic, adherence to non-pharmaceutical interventions and masking has generally been low.^8–10^ Similarly, vaccination uptake may be lower where there is limited perceived danger from COVID-19, and concerns about new COVID-19 vaccines may outweigh fear of the disease.^3,11–14^

With the increasing availability of COVID-19 vaccines in many low-and-middle-income countries (LMIC), understanding the expected acceptability of COVID-19 vaccination and challenges to uptake is critical. Through a survey nested within a measles-rubella mass vaccination campaign, parents were asked a set of questions to gauge their knowledge, attitudes, and beliefs about COVID-19 and COVID-19 vaccination. We present an analysis to assess the intent to vaccinate for caregivers and children, and how these correlated with knowledge and concerns of COVID-19 disease and vaccines.

## Methods

We nested a Measles and Rubella serosurvey in the November 2020 MR vaccination campaign embedded in Child Health Week at 30 sites (20 fixed sites and 10 outreach sites) in two districts in Zambia (Supplementary Material 1). First, Choma district in Southern Province is primarily rural with a projected population of 303,533.^15^ In 2019, the MCV1 coverage for Choma district was 90%. The second, Ndola district in Copperbelt Province is primarily urban with a projected population of 585,974. The MCV 1 coverage in 2019 was 87%. Ethical approval was obtained from the Tropical Diseases Research Center ethics review committee and the Johns Hopkins Institutional Review Board. Further regulatory approval was given by the Zambia National Health Research Authority.

Demographic and vaccination history data were collected using a standardized questionnaire on a tablet-based application (Supplementary Material 2). We included questions on perceptions of COVID-19 disease and the vaccine designed to capture perceived benefits, barriers, and threats from the Health Belief Model with the intent to vaccinate their child and themselves.^16^ Response choices for all questions were yes, no, or do not know. The two intent-to-vaccinate statements were phrased in reverse, asking refusal to vaccinate self versus acceptance for the child.

We present descriptive analyses of the COVID-related questions, overall and stratified by district. Chi-square analyses compared the relationships between vaccine safety and efficacy as well as perceived threat and severity. To evaluate the relationship between intent to vaccinate and the perception of COVID-19 disease or vaccine, log binomial regression models were fit, adjusting for the other health belief constructs. Campaign daily summaries of the perceptions of COVID-19 disease and vaccine were calculated for each facility and plotted against similar summaries with intent to receive vaccination. Coefficients of determination were calculated for each relationship.

## Results

From 23–29 November 2020, we enrolled 2,400 children between the age of 9 months and 5 years who were brought to vaccination sites in two districts in Zambia. All received a campaign dose of MR vaccine prior to the survey. All responses were from adult caregivers.

Overall, 92% of the caregivers reported that they intended to have their child vaccinated against COVID-19, but only 66% reported planning to receive the vaccine themselves. Caregivers in Ndola reported significantly higher willingness than those residing in Choma to both vaccinate their child (97% vs 86%; *p* < .001) and receive the vaccine themselves (85% vs 47%; *p* < .001; Supplementary Material 3).

**Figure 1.**
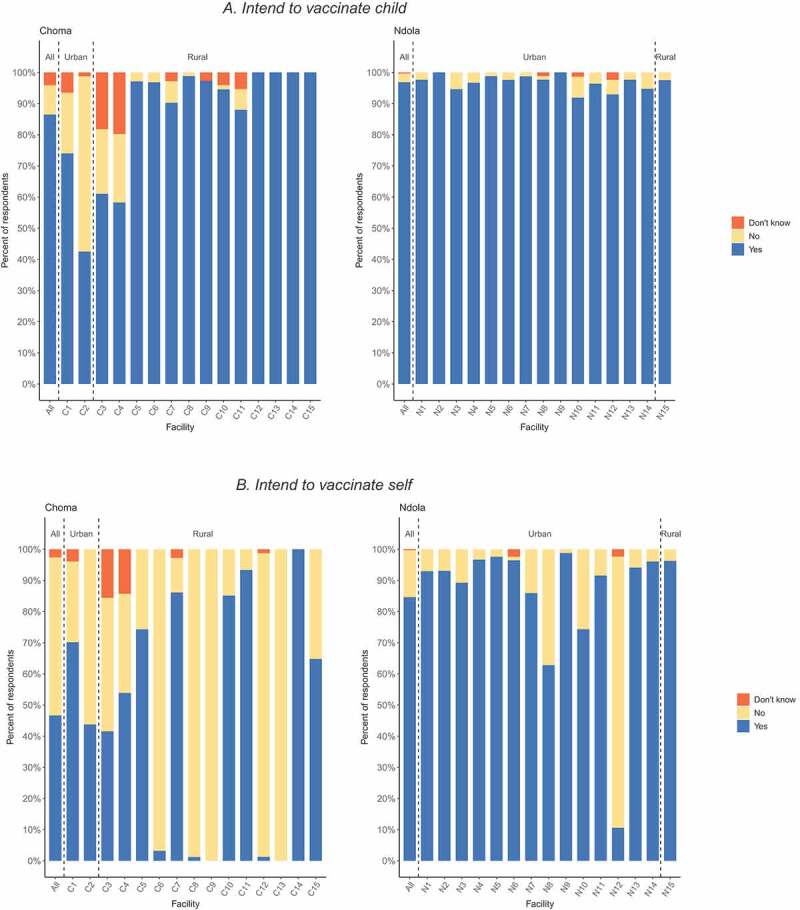
Caregivers’ intent to vaccinate their child (a) and receive the vaccine (b) against COVID-19 by district and campaign setting, among caregivers attending supplemental immunization activity in Zambia. Facilities designated as C3 and C4 represent peri-urban settings.

A high willingness to vaccinate their child in Ndola was observed across all 15 campaign facilities (Figure 1(a)). However, in Choma, four (27%) campaign facilities, serving urban or peri-urban areas, had higher degrees of vaccine hesitancy, with caregivers 12.8 times (95% CI, 8.7–18.9) more likely to express concerns toward vaccinating their children compared to rural facilities. No differences between urban and rural sites were observed in Ndola. In both districts, maternal education was higher at urban sites compared to rural sites (Supplementary Material 4).

Caregivers’ hesitancy to receive a vaccine was observed at most facilities in Choma, with five (33%) facilities having less than 5% of the caregivers intending to receive the vaccine (Figure 1(b)). In Ndola, only one facility had high vaccine hesitancy among caregivers.

**Figure 2.**
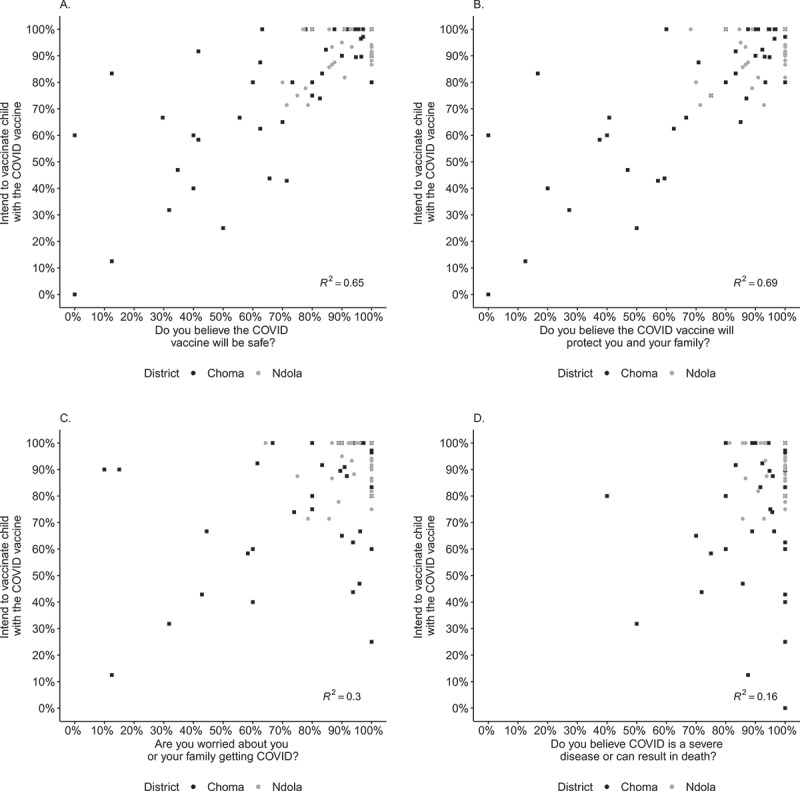
Relationship between intent to vaccinate child and perceptions of disease and vaccine among caregivers attending supplemental immunization activity in Zambia. Campaign daily summaries of the perceptions of COVID-19 disease and vaccine were calculated for each facility then plotted against similar summaries for intent to vaccinate child.

Perceptions about vaccine safety and efficacy were the strongest predictors of vaccine acceptance, for both adult and child vaccination. Overall, 89% of the caregivers thought the vaccine was safe and 91% thought it was effective, with a high concordance between belief in vaccine safety and efficacy (Pearson’s r = 0.93, Figure 2(a,b)). Among caregivers who believed the vaccine was safe or effective, 67% were willing to be vaccinated themselves, and 97% were willing to vaccinate their children (Figure 2(a,b)).

In multivariate logistic regression, controlling for beliefs regarding vaccine efficacy and perceived disease susceptibility and severity, those who believed COVID-19 vaccines would be safe were 40% more likely (RR = 1.40; 95% CI, 1.20–1.62) to report intent to vaccinate their children compared to those who were unsure or did not believe it would be safe (Table 1). Similarly, caregivers who believed COVID-19 vaccines would be effective were 77% more likely (RR = 1.77; 95% CI, 1.45–2.15) to report intent to vaccinate their children. Caregivers who believed the vaccine was effective were 31% more likely (RR = 1.31; 95% CI, 1.09–1.57) to accept the vaccine for themselves.

**Table 1.** Intent to vaccinate child and self, controlling for Health Belief Model constructs. Relative risks of intent to vaccinate a child (“Child”) or self (“Adult”) among caregivers seeking measles and rubella vaccination for their child at the Zambian Child Health Week, Choma and Ndola Districts, November 2020. These relative risks are unadjusted and adjusted for other Health Belief Model constructs

	Choma		Ndola		Overall	
	Unadjusted	Adjusted	Unadjusted	Adjusted	Unadjusted	Adjusted
Child						
safety	**2.18 (1.88, 2.52)**	**1.35 (1.16, 1.59)**	**1.85 (1.41, 2.43)**	-	**2.13 (1.87, 2.43)**	**1.40 (1.20, 1.62)**
efficacy	**2.58 (2.14, 3.10)**	**1.91 (1.53, 2.39)**	**1.82 (1.35, 2.45)**	-	**2.43 (2.07, 2.85)**	**1.77 (1.45, 2.15)**
threat	**1.48 (1.31, 1.68)**	1.02 (0.97, 1.08)	**1.35 (1.10, 1.66)**	-	**1.49 (1.34, 1.66)**	1.04 (0.98, 1.09)
severity	**2.07 (1.56, 2.76)**	1.26 (0.99, 1.59)	**1.35 (1.06, 1.73)**	-	**1.82 (1.49, 2.24)**	1.13 (0.97, 1.30)
Adult						
safety	0.89 (0.77, 1.03)	0.81 (0.66, 1.00)	**1.54 (1.19, 2.01)**	1.04 (0.81, 1.32)	**1.30 (1.15, 1.46)**	1.02 (0.88, 1.18)
efficacy	0.98 (0.83, 1.15)	1.23 (0.98, 1.54)	**1.87 (1.32, 2.65)**	**1.68 (1.12, 2.52)**	**1.43 (1.24, 1.64)**	**1.31 (1.09, 1.57)**
threat	**0.81 (0.69, 0.94)**	**0.71 (0.60, 0.85)**	**1.62 (1.19, 2.21)**	**1.45 (1.07, 1.97)**	**1.20 (1.06, 1.37)**	0.96 (0.85, 1.09)
severity	**1.88 (1.23, 2.89)**	**2.25 (1.46, 3.47)**	1.33 (0.99, 1.79)	1.03 (0.82, 1.29)	**1.84 (1.39, 2,43)**	**1.63 (1.23, 2.17)**

Relative risks are presented here unadjusted and adjusted for other health belief model constructs. Values in bold are statistically significant. Ndola child model (-) failed to converge.

Overall, most caregivers believed COVID-19 is a severe disease (96%) and were worried about themselves or their family getting COVID-19 (92%). Among those worried about getting COVID-19 or those who thought COVID-19 is a severe disease, 67% reported they would accept the vaccine for themselves, and 93% would vaccinate their children (Figure 2(c,d)).

After controlling for beliefs around safety and effectiveness, caregivers who believed COVID-19 disease was severe were significantly more likely to report intent to accept the vaccine for themselves (RR = 1.63, 95% CI, 1.23–2.17) but not for their children (RR = 1.13, 95% CI,0.97–1.30). Caregivers who were concerned about getting COVID-19 were not significantly more likely to report intent to receive the vaccine themselves or their children (Table 1).

Only 14% of the caregivers willing to vaccinate their child reported they would refuse for themselves in Ndola (Supplementary Material 5A). However, this increased to 50% of the caregivers residing in Choma, concentrated at five facilities (\Supplementary Material 5B). Those willing to accept vaccination for their child but not themselves had similar perceptions of disease and vaccine as those willing to accept vaccine for both child and self (Supplementary Material 6). Those willing to receive the vaccine but not vaccinate their child were more concerned about the disease than those not willing to accept the vaccine at all, but over 50% expressed concerns about vaccine safety and efficacy.

## Discussion

Through a survey of caregivers bringing children to an MR vaccination campaign, we found a high acceptance of the COVID-19 vaccine for children, likely translating from familiarity and high acceptance rates for other childhood vaccines. However, the acceptance of the COVID-19 vaccine among adults may face challenges even in this population that is highly willing to vaccinate their children. This is important as adults, particularly health workers are the primary targets for the first COVID-19 vaccines and are at increased risk for COVID-19 disease.

As vaccine rollout among LMICs like Zambia begins, careful consideration of the challenges of vaccine uptake among adults is critical. Most LMICs plan to distribute COVID-19 vaccine through mass vaccination campaigns.^17^ While many have experience with mass vaccination campaigns targeting children, their experience in administering vaccines to adults is limited.

This survey provides a glimpse of the potential factors contributing to an individual’s vaccine acceptance. Within our study population, vaccine safety and efficacy were the strongest predictors of intent to vaccinate children. This relationship between the perceived benefit of protection from the vaccine and the intent to vaccinate is well documented for other vaccines.^12^ Similarly, belief in the safety of vaccines has been an important motivator for the intent to vaccinate in countries currently rolling out COVID-19 vaccines.^13,14^ In Choma, caregivers from urban sites were less likely to report intending to vaccinate their children than those from rural sites. This could be resulting from differences in maternal education or socioeconomic status, which have been associated with vaccine hesitancy.^18,19^ Although we did not investigate this, one plausible explanation for this unusual finding is that there has been a lot of misinformation and miscommunication about the safety of COVID-19 vaccines online. Urban populations generally have more access to the internet and social media, and this may have impacted their beliefs on COVID-19 vaccines. Online misinformation is predictive of the belief that vaccines are unsafe and associated with lower vaccination coverage.^20^

Among adults, the strongest predictor of vaccine intent was the perception of COVID-19 disease severity followed by the efficacy of the vaccine. These findings indicate important targets for vaccination education and communication efforts. Access to news and social media, particularly among higher-educated individuals, may influence willingness to accept the COVID-19 vaccine.^20,21^ Providing messages focused on the severity of COVID-19 and vaccine effectiveness through traditional sources of information, such as healthcare personnel in addition to social media could encourage uptake among adults.

Previous studies have also found that caregivers might be willing to vaccinate their children but not themselves. Oral cholera vaccine (OCV) coverage in Lusaka following a mass vaccination campaign in 2016 was considerably lower among adults than children, even after a catch-up campaign targeting adults.^22^ Globally, coverage rates are substantially lower for adult vaccines than childhood vaccinations.^23^ Agreeing to vaccinate a child likely reflects confidence in the vaccine, while refusal to self may reflect a lower perceived risk to personal health. Targeted messaging will be required to capture a population less familiar, and possibly less comfortable, with receiving vaccines.

While this study highlights the critical findings and potential challenges relevant to COVID-19 vaccine uptake in Zambia, it also faces several limitations. First, this study population represented a group of caregivers willing to accept other vaccines for their children (i.e., the MR vaccine) and thus may represent an optimistic outlook of COVID-19 vaccine acceptance. Furthermore, this study was conducted in November 2020, at a point at which COVID-19 had not substantially impacted Zambians, with only 1,300 cumulative cases and less than 50 cases reported per day. In the 4 months since this study, the outbreak has increased substantially, exceeding 1,500 cases reported daily in January.^8^ Perceptions of disease severity and risk to oneself and one’s family may have changed with the progression of the pandemic, and as a result, the relationship between perception of risk and vaccine acceptance may be stronger now.^9,24^Additionally, because the vaccine acceptance questions for children and adults were asked in reverse, there may have been some misinterpretation of the question for some individuals.

We are also limited in our ability to delve deeper into the reasons behind hesitancy. This survey capitalized on the opportunity to engage caregivers at the point of service delivery. Although questions were limited, they captured multiple aspects of the Health Belief Model, a framework used to define individual factors for health behaviors.^16^ We were unable to probe for additional details or underlying factors influencing perceptions and intentions. Differences between districts, among urban populations, or the role of caregiver education in intent to vaccinate a child are also difficult to interpret in the absence of additional details that may have influenced the caregivers’ decisions.

Our findings and limitations highlight the need for future qualitative research to understand the underlying motivators and concerns among those who are and are not intending to receive a COVID-19 vaccine. Future research is needed to reevaluate COVID-19 vaccination intent under the current epidemiological situation and delve deeper into the underlying barriers to receiving the vaccine. These are especially needed among adults, who will be the initial targets of the COVID-19 vaccination efforts. Through this and other studies, we can help inform both the content and strategy of targeted messaging to ensure high vaccine uptake.

## Conclusions

While Zambia has high childhood vaccination coverage, vaccine hesitancy, particularly among adults, may present serious challenges for COVID-19 vaccination. In other populations where general vaccination acceptance and coverage are lower, these challenges might be even greater. Given the reported vaccine hesitancy and misinformation throughout the pandemic, providing accurate information through trusted sources of information will be critical to increasing vaccine demand.^13^ Simplified assumptions of COVID-19 dynamics suggest that between 50% and 67% of the population will need to be vaccinated to control the spread of the virus. Reported levels of vaccination acceptance among adults in these populations threatens the success of efforts to control the disease. National vaccine deployment plans should include community sensitization tailored for the adult population.

## Supplementary Material

Supplemental Material
