# Supplementary material for: COVID-19 vaccine hesitancy in Zambia: a glimpse at the possible challenges ahead for COVID-19 vaccination rollout in sub-Saharan Africa
Source: Hum Vaccin Immunother. 2021 Jul 6;18(1):1–6. doi: 10.1080/21645515.2021.1948784 (PMC8920139; doi:10.1080/21645515.2021.1948784)

**Supplementary Material 1, Map. Map of survey setting and measles-rubella campaign vaccination sites**

1. Map of Zambia indicating districts where survey took place (Ndola District in Copperbelt Province and Choma District in Southern Province).


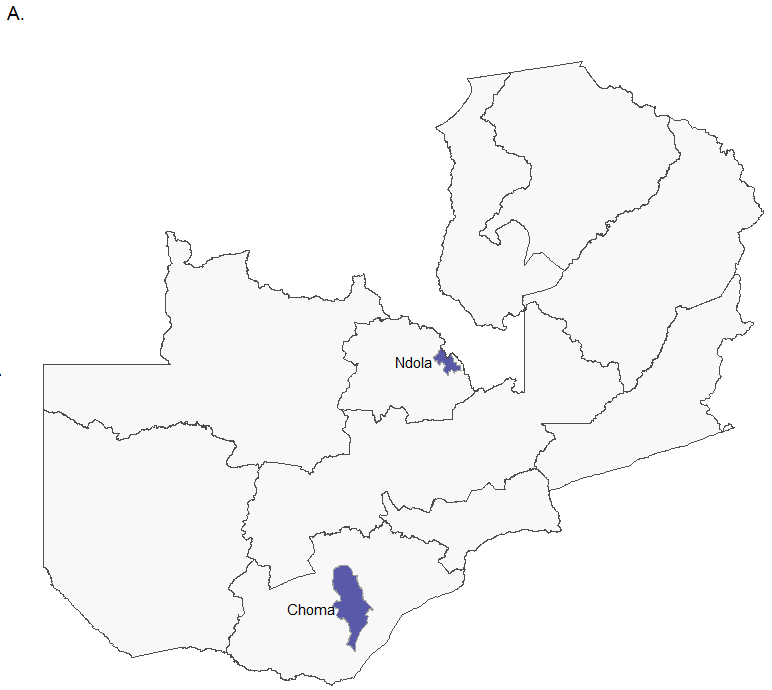


B. Measles-rubella campaign vaccination sites by district.  For campaign sites indicated in blue the survey was conducted at the fixed campaign site (health facility) and outreach campaign sites (2 survey sites per facility).  The survey was conducted in a total of 15 campaign sites per district (30 total between the two districts).
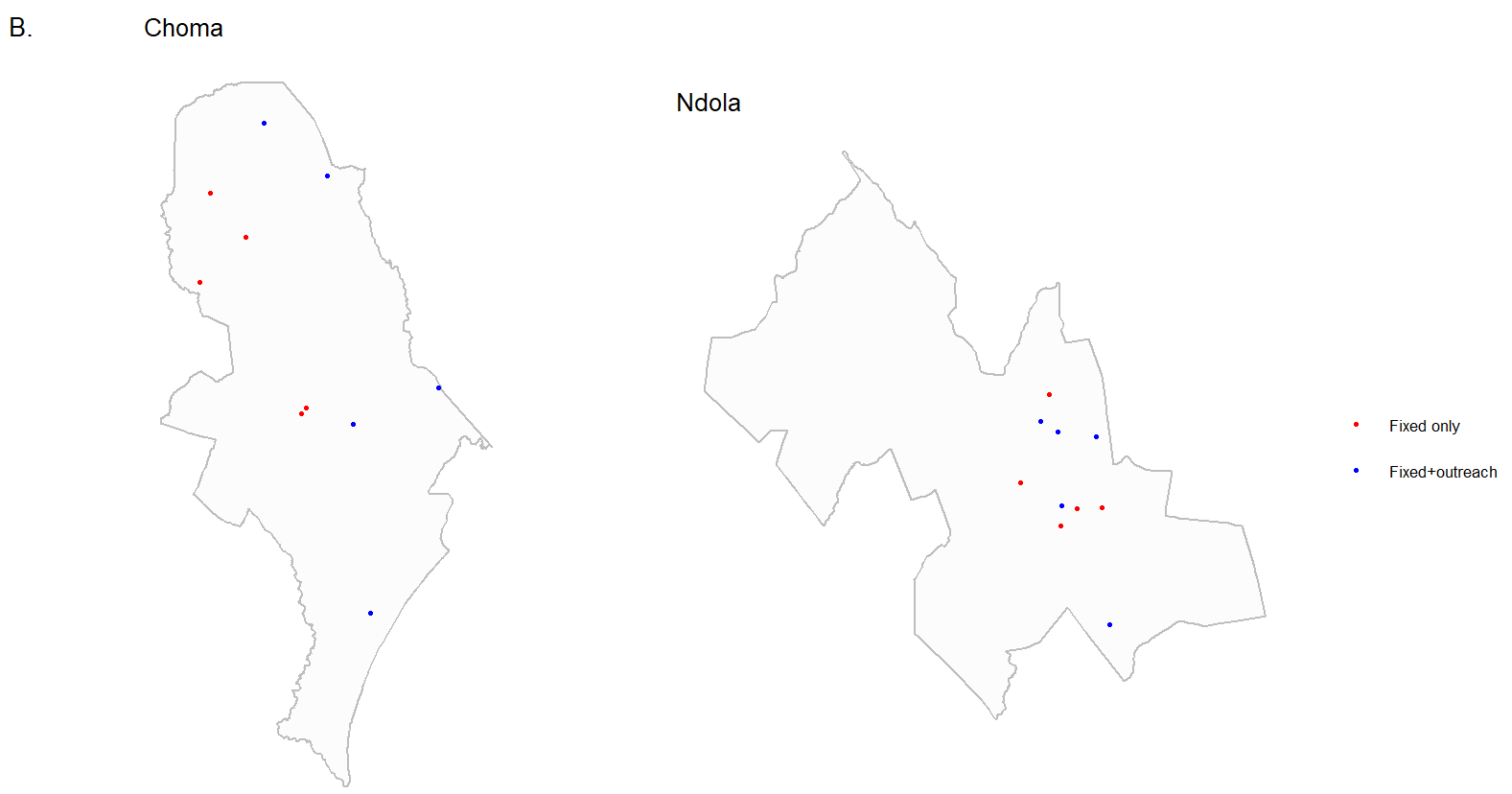

Supplement: Supplemental Material [file KHVI_A_1948784_SM8910.zip › ZambiaCOVID_SupplementaryMaterial1_Map.docx]
