# Supplementary material for: COVID-19 vaccine hesitancy in Zambia: a glimpse at the possible challenges ahead for COVID-19 vaccination rollout in sub-Saharan Africa
Source: Hum Vaccin Immunother. 2021 Jul 6;18(1):1–6. doi: 10.1080/21645515.2021.1948784 (PMC8920139; doi:10.1080/21645515.2021.1948784)
Supplement: Supplemental Material [file KHVI_A_1948784_SM8910.zip › ZambiaCOVID_SupplementaryMaterial1_Questionnaire.docx]

| Child Form (9 months - 5 years) | | | | |
| --- | --- | --- | --- | --- |
| *This questionnaire is to be administered to parents/guardians of children aged 9 months-5 years who were present at the time of the campaign and selected for the survey. Confirm parent/guardian has signed the permission form before administering the survey.* | | | | |
| Coordinating Center | 1-Macha  2-TDRC | | | |
| Site Code | (drop down menu with options specific to the site) | | | |
| Participant ID | ___ ___ ___ ___ ___ ___ ___ | | | |
| 1. Type of campaign site | Fixed site……….1  Outreach site…2 | | | |
| 2. Date of interview | __ __ / __ __ / __ __ __ __  D D M M Y Y Y Y | | | |
| Time of interview (autopopulated) |  | | | |
| 3. Did respondent provide parental permission for child to participate in the study? | Yes ……………………………………………………..………1  No, refused due to blood collection …….….….2  No, refused due to other reason ………………..3  No, caregiver not present………………………..….4  Other, specify………………………………….…………88  Specify: ___________________  *If response is Yes, then continue with survey. If not, do not collect any more data. Stop interview and thank family.* | | | |
| 4. Are you the person who regularly takes the child to their health visits? | Yes…………….1  No….…………0 | | | |
| 5.Child’s gender | Male…………….1  Female…………0 | | | |
| 6a. Do you know the date of birth for the child?  Yes …………………………………..………………1  No ..………………………………….………….….0 | 6b. Date of birth  __ __ / __ __ / __ __ __ __  D D M M Y Y Y Y  *Record information from the Under 5 Card if available. If month known but day unknown record “15” for day. If month and/or year unknown go to Q6c to record age.* | | | |
| 6c. If month and/or year of birth is not known, how old is child? *If less than 1 year record in months.*  Age Unit: 1 – Months, 2 – Years  Age __ __ | | | | |
| **COVID-19** | | | | |
| *We will now read you a few questions about COVID-19 and a future COVID-19 vaccine. There is currently no vaccine available for COVID-19, but we would like to get a better understanding of your view of the vaccine for when one becomes available.* | | | | |
|  | | **Yes** | **No** | **Don’t know** |
| Are you worried about you or your family getting COVID? | | **1** | **0** | **99** |
| Do you believe COVID is a severe disease or can result in death? | | **1** | **0** | **99** |
| Will you refuse the COVID vaccine when available? | | **1** | **0** | **99** |
| Will you have your child vaccinated with the COVID vaccine when available? | | **1** | **0** | **99** |
| Do you believe the COVID vaccine will be safe? | | **1** | **0** | **99** |
| Do you believe the COVID vaccine will protect you and your family against COVID? | | **1** | **0** | **99** |
| Was your child’s routine vaccination delayed because of the pandemic? | | **1** | **0** | **99** |
