# Supplementary material for: COVID-19 vaccine hesitancy in Zambia: a glimpse at the possible challenges ahead for COVID-19 vaccination rollout in sub-Saharan Africa
Source: Hum Vaccin Immunother. 2021 Jul 6;18(1):1–6. doi: 10.1080/21645515.2021.1948784 (PMC8920139; doi:10.1080/21645515.2021.1948784)
Supplement: Supplemental Material [file KHVI_A_1948784_SM8910.zip › ZambiaCOVID_SupplementaryMaterial2_Table.docx]

**Supplementary Data 2, Table. Intent to vaccinate child and self among caregivers seeking measles-rubella vaccine for child during mass vaccination campaign, November 2020, Zambia.**

|  | Choma (N=1197)  *n (%)* | Ndola (N=1203)  *n (%)* |
| --- | --- | --- |
| Intend to vaccinate child |  |  |
| Yes | 1035 (86.5) | 1165 (96.8) |
| No | 113 (9.4) | 34 (2.8) |
| Don’t know | 49 (4.1) | 4 (0.3) |
| Intend to receive vaccine |  |  |
| Yes | 559 (46.7) | 1018 (84.6) |
| No | 607 (50.7) | 181 (15.0) |
| Don’t know | 31 (2.6) | 4 (0.3) |
