# Supplementary material for: COVID-19 vaccine hesitancy in Zambia: a glimpse at the possible challenges ahead for COVID-19 vaccination rollout in sub-Saharan Africa
Source: Hum Vaccin Immunother. 2021 Jul 6;18(1):1–6. doi: 10.1080/21645515.2021.1948784 (PMC8920139; doi:10.1080/21645515.2021.1948784)
Supplement: Supplemental Material [file KHVI_A_1948784_SM8910.zip › ZambiaCOVID_SupplementaryMaterial3_Table.docx]

**Supplementary Material 3, Table. Discrepancy between intent to vaccinate children versus adults**

**A. Ndola**

|  | | Intend to vaccinate child | | | |
| --- | --- | --- | --- | --- | --- |
|  |  | No | Yes | Don’t know | Total |
| Intend to receive vaccine | No | 18 | 162 | 1 | 181 |
|  | Yes | 16 | 1001 | 1 | 1018 |
|  | Don’t know | 0 | 2 | 2 | 4 |
|  | Total | 34 | 1165 | 4 | 1203 |

**B. Choma**

|  | | Intend to vaccinate child | | | |
| --- | --- | --- | --- | --- | --- |
|  |  | No | Yes | Don’t know | Total |
| Intend to receive vaccine | No | 80 | 517 | 10 | 607 |
|  | Yes | 28 | 507 | 24 | 559 |
|  | Don’t know | 5 | 11 | 15 | 31 |
|  | Total | 113 | 1035 | 49 | 1197 |
