# Supplementary material for: COVID-19 vaccine hesitancy in Zambia: a glimpse at the possible challenges ahead for COVID-19 vaccination rollout in sub-Saharan Africa
Source: Hum Vaccin Immunother. 2021 Jul 6;18(1):1–6. doi: 10.1080/21645515.2021.1948784 (PMC8920139; doi:10.1080/21645515.2021.1948784)
Supplement: Supplemental Material [file KHVI_A_1948784_SM8910.zip › ZambiaCOVID_SupplementaryMaterial4_MaternalEducationTable.docx]

Supplementary Material 4, Table. Maternal education by campaign site setting and district

| Mother’s education | Choma | | Ndola | |
| --- | --- | --- | --- | --- |
|  | Rural | Urban^a^ | Rural | Urban |
| Total | 872 | 325 | 80 | 1123 |
|  | n (%) | n (%) | n (%) | n (%) |
| None | 41 (4.7) | 5 (1.5) | 4 (5.0) | 44 (3.9) |
| Primary | 550 (63.1) | 122 (37.7) | 38 (47.5) | 318 (28.3) |
| Secondary | 259 (29.7) | 148 (45.7) | 37 (46.3) | 679 (60.5) |
| Higher than secondary | 21 (2.4) | 49 (15.1) | 1(1.3) | 81 (7.2) |

1. The two peri urban campaign sites in Mochipapa are treated s urban in this analysis.
