# Supplementary material for: COVID-19 vaccine hesitancy in Zambia: a glimpse at the possible challenges ahead for COVID-19 vaccination rollout in sub-Saharan Africa
Source: Hum Vaccin Immunother. 2021 Jul 6;18(1):1–6. doi: 10.1080/21645515.2021.1948784 (PMC8920139; doi:10.1080/21645515.2021.1948784)
Supplement: Supplemental Material [file KHVI_A_1948784_SM8910.zip › ZambiaCOVID_SupplementaryMaterial6_Figure.pdf]

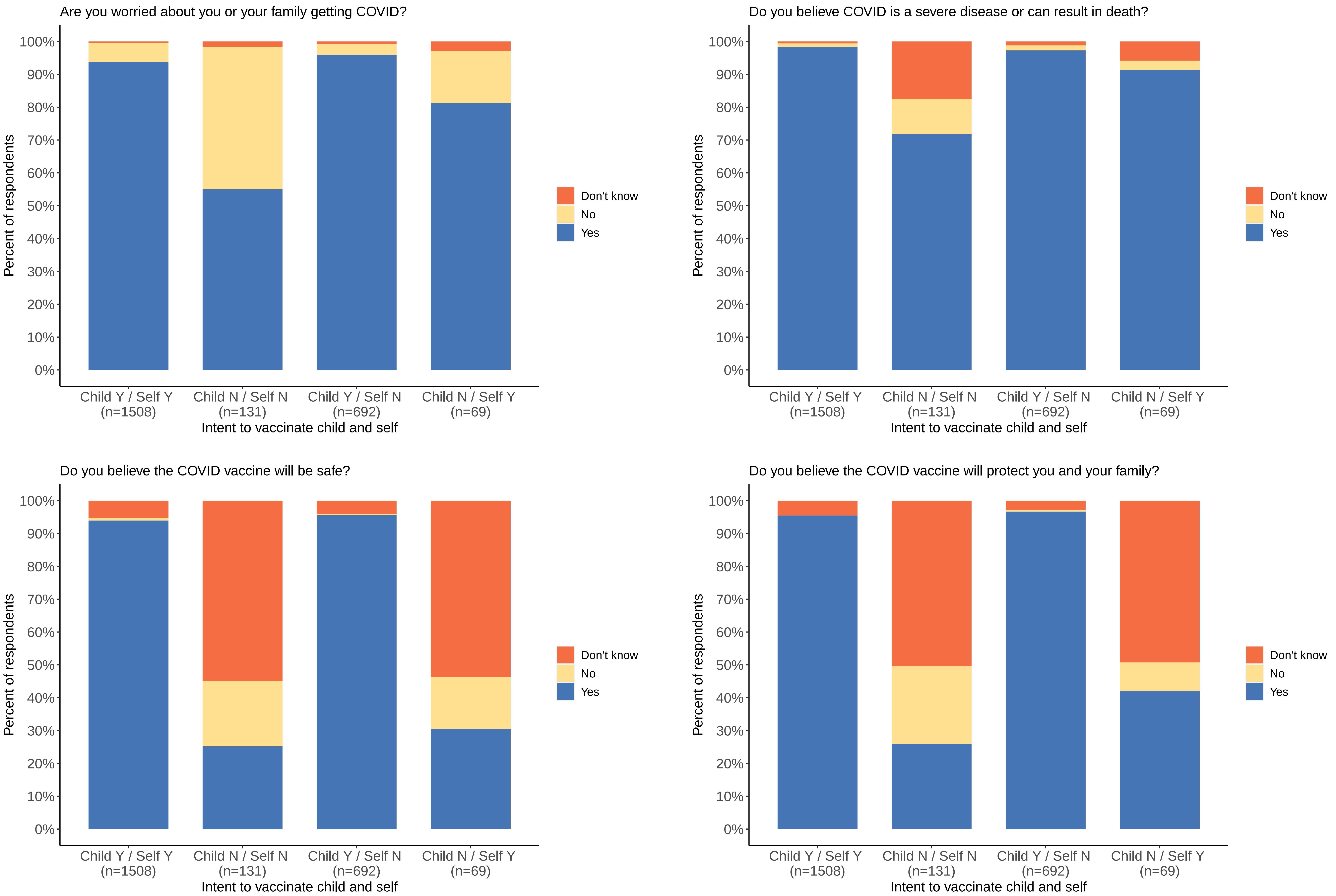
Are you worried about you or your family getting COVID?
Do you believe COVID is a severe disease or can result in death?
Do you believe the COVID vaccine will be safe?
Do you believe the COVID vaccine will protect you and your family?
Percent of respondents
100%
90%
80%
70%
60%
50%
40%
30%
20%
10%
0%
Don't know
No
Yes
Child Y / Self Y (n=1508)
Child N / Self N (n=131)
Child Y / Self N (n=692)
Child N / Self Y (n=69)
Intent to vaccinate child and self
